# Digital play addiction tendency in 5- to 6-year-old children: the role of parental mediation strategies in Russia and Türkiye

**DOI:** 10.3389/fpsyg.2026.1898639

**Published:** 2026-07-15

**Authors:** Olga Almazova, Nesrin Isikoglu, Nebojsa Cokorilo, Patrik Drid

**Affiliations:** 1 Laboratory of Childhood Psychology and Digital Socialization, Federal Scientific Center for Psychological and Interdisciplinary Research, Moscow, Russia; 2 Early Childhood Education, Pamukkale University, Denizli, Türkiye; 3 Faculty of Sport and Physical Education, University of Novi Sad, Novi Sad, Serbia; 4 Faculty of Medicine, University of Novi Sad, Novi Sad, Serbia

**Keywords:** cross-cultural comparison, digital play addiction, parental mediation, preschoolers, Russia, Turkey

## Abstract

**Introduction:**

The rapid digitalization of early childhood has raised growing concerns about the development of digital play addiction among preschoolers. However, little is known about how parental mediation strategies relate to this tendency across different cultural contexts. To address this gap, this study had two primary aims: (1) to identify similarities and differences in the tendency toward digital play addiction among 5- to 6-year-old children and in parental digital mediation strategies between Russia and Turkey, and (2) to examine the relationship between these mediation strategies and children’s addiction tendency in both countries.

**Methods:**

The sample consisted of 452 parents of 5- to 6-year-old children (226 from Russia and 226 from Turkey). All participants completed the Digital Play Addiction Tendency Scale (DPAT) and the Digital Play Parental Mediation Scale (DPPM). Latent mean comparisons were conducted to examine cross-cultural differences in parental mediation practices, and regression analyses were performed to identify predictors of addiction tendency subscales in each country.

**Results:**

Latent mean comparisons revealed that Turkish parents showed a significantly lower latent threshold mean on the Encouraging dimension compared to their Russian counterparts. No statistically significant cross-cultural differences were found for the Active, Permissive, or Technical mediation dimensions, indicating a relatively uniform structural profile across both countries in how these three positive strategies are hierarchically deployed. Regression analyses showed that Encouraging Parental Mediation was a consistent positive predictor of most addiction tendency subscales in both countries. However, notable cross-cultural differences emerged: in Russia, Constant Play was positively associated with both Encouraging and Permissive Mediation but negatively linked to Active Mediation, whereas in Turkey, Encouraging Mediation was the dominant predictor across all subscales.

**Discussion:**

These findings suggest that while the overall profile of parental mediation strategies is similar across Russia and Turkey, the specific relationships between mediation practices and children’s addiction tendency exhibit both culturally universal and culturally specific patterns. The protective role of Active Parental Mediation observed in Russia highlights the importance of discussing digital content with children. The results have practical implications for developing evidence-based parental guidance programs that are tailored to the specific cultural contexts of each country.

## Introduction

From early childhood, children engage with digital devices (DDs) (Barr et al., 2024; Soldatova et al., 2024). By the age of 5–6 years, children’s screen time reaches approximately 2 hours per day. Passive screen time (watching video content) is, on average, twice as high as active screen time (interactive engagement with digital devices, primarily digital games) (Lakicevic et al., 2025; Chichinina et al., 2024). At 5–6 years old, before entering school, children’s use of digital devices is typically controlled and limited by parents or other close adults (Milosevic et al., 2022; Veraksa et al., 2024).

Although active screen time remains substantially lower than passive screen time during the opreschool years, the impact of digital games on child development has become a central focus for many researchers (Belova and Shumakova, 2025; Işıkoğlu et al., 2025; Ngyah-Etchutambe, 2025). More than 80% of children aged 5–6 years already have experience with digital games (Chichinina et al., 2024), and play, in general, represents a highly significant type of activity (Gavrilova et al., 2025; Veresov and Veraksa, 2022). Despite considerable research on the effects of screen time (Brauchli et al., 2024a; Gath et al., 2023), game types (Plotnikova et al., 2023), and parental involvement in children’s digital activities (Almazova et al., 2025; Radesky et al., 2023) on emotional, cognitive, and regulatory development, far less attention has been paid to identifying tendencies toward digital game addiction in children of this age and the factors that may influence the formation of such tendencies (Budak and Işıkoğlu, 2022; Güneş, 2022).

When examining the digital activity of preschoolers in Russia and Turkey, both common and distinct features emerge. Common features include: (1) digital games represent an important and highly attractive type of digital activity for children (Işıkoğlu et al., 2025; Chichinina et al., 2024); (2) preschool children most often do not own personal digital devices (Almazova et al., 2025; Proekt et al., 2024), (3) in both Turkey and Russia, 5- to 6-year-olds attend kindergarten rather than school. Differences include: (1) within the educational process, including preschool institutions in Turkey, children are quite often taught to use smartboard and tablets for learning and play, a practice that remains rare in Russia (Kahriman and Borg, 2024; Kholodova et al., 2025); (2) in most public kindergartens in Turkey, children attend only half a day on weekdays, whereas in Russia they attend full-day programs (Özgan, 2010; Veraksa et al., 2023); (3) the percentage of children attending preschool differs between the two nations, with approximately 93% enrollment in Russia compared to nearly 80% in Turkey (Chichinina et al., 2024; Işıkoğlu et al., 2025; Millî Eğitim Bakanlığı (MEB), 2025). This combination of similarities and differences provided a unique opportunity to examine how contextual factors may influence digital engagement patterns in preschool children.

### Tendency toward digital play addiction in 5- to 6-year-old children

Digital play addiction is defined as “the excessive, irregular, or uncontrollable use of a device by an individual that disrupts his or her normal daily life and interferes with the task at hand” (Lemmens et al., 2009, p.78). In the context of children, it is customary to speak not of addiction per se, but of a *tendency toward* addiction. Drawing on the DSM-5, researchers have identified the following aspects as important for identifying the tendency toward digital play addiction in children and adolescents: (1) play becomes a significant activity, influencing a person’s emotions, thoughts, and behavior; (2) an increase in the duration of play; (3) a negative impact of gaming involvement on relationships with others; (4) negative emotions and behavior when deprived of digital play (Lemmens et al., 2009; Kesicioğlu, 2026).

Regarding children aged 5–6 years, their self-regulation is insufficiently developed for them to independently cease digital play activities. Consequently, researchers argue that the tendency toward digital play addiction is more characteristic of children than of adults (Schulz van Endert, 2021; Rosendo-Rios et al., 2022). It is important to note, however, that the control mechanism that prevents a tendency from developing into a full-fledged addiction may develop in a child as they mature (Vazsonyi and Huang, 2010).

### Digital parental mediation

Children typically begin using digital devices within the family setting. Parents and other close adults play a significant role in how a child begins to master digital devices (Barr et al., 2024; Chaudron et al., 2018), which necessitates the study of strategies that parents employ when introducing children to digital devices in order to ensure safety and minimize harm. Parental mediation, in the context of children’s use of digital devices, refers to actions by parents aimed, on the one hand, at establishing and enforcing certain rules (regulation) and, on the other hand, at jointly discussing with children their activity in the digital environment (Livingstone et al., 2017). Despite the wide variety of classifications of these strategies, they can be divided into two groups: parental control strategies and parental support strategies (Eastin et al., 2006).

Parental control strategies are subdivided into: (a) technical control – the use of special programs that block undesirable content; (b) monitoring of the child’s digital activity; and (c) rules and restrictions concerning screen time, content, and types of activities (Kuldas et al., 2024; Symons et al., 2017).

Parental support strategies include: (a) mediation, in which the parent discusses content with the child, encouraging the use of positive content and discouraging the use of negative content; (b) stimulation, which involves the purposeful development of the child’s digital skills; (c) safety assurance, through which parents discuss with the child the rules for safe Internet use, thereby developing the child’s digital hygiene (Kurgansky et al., 2023; Sciacca et al., 2022; Soldatova and Teslavskaya, 2019).

### The relationship between parental digital mediation and children’s tendency toward digital play addiction

The effectiveness of parental mediation strategies for ensuring children’s safe use of digital devices and minimizing the negative impact of digital devices on child development has been examined in a number of studies. Some studies show that the use of parental control strategies protects children from certain digital risks and leads to a reduction in screen time (Beyens et al., 2018; Fardouly et al., 2018), while others note that it is important not only to control but also to explain to the child the reasons for the rules and the consequences of prohibited behavior (Dias et al., 2016; Nikken, 2017) – an approach largely implemented in parental support strategies. It is precisely parental support strategies that allow children to be more critical of digital content and to develop digital competence more broadly (Almazova et al., 2025; Schmuck, 2021; Wang et al., 2023).

Despite the apparent effectiveness of strategies belonging to the parental support group, the strategy of *encouraging* children’s digital activity remains debatable in terms of its positive impact (Keya et al., 2020). If a parent pushes a child to use digital devices (especially digital games) when the child is bored or in order to free up their own time, this may lead to the formation of digital play addiction (Budak and Işıkoğlu, 2022).

Parents often experience difficulties in accompanying their children’s digital activity, especially play activity, which reveals the need for educational initiatives that include a description of more and less effective digital mediation strategies (Klopotova et al., 2022; Spasskaya et al., 2023).

A separate risk factor for the development of a tendency toward digital play addiction is allowing a preschool-aged child to use digital devices independently without adequate adult supervision (Işıkoğlu et al., 2025). Although such permissive parental behavior is not traditionally classified as a parental mediation strategy, identifying the role of parents in the development of a tendency toward digital play addiction requires taking into account the extent of such parental behavior.

Parents may combine different strategies belonging to different groups (Beyens et al., 2018; Lim, 2016). Therefore, it is important to assess the degree of use of each strategy and to identify the contribution of different strategies to the tendency toward digital play addiction in preschoolers (Kuldas et al., 2024; Neumann et al., 2018).

### Current research

Although the relationship between parental digital mediation strategies and children’s digital immersion and patterns of digital device use is well established (Rosendo-Rios et al., 2022; Rudnova et al., 2023), identifying the specific features of the tendency toward digital addiction in preschool children and determining the role (whether protective or facilitative) of individual parental digital mediation strategies in the formation of this tendency remains an understudied issue (Işıkoğlu et al., 2025). Furthermore, little is known about whether these relationships are culturally universal or shaped by broader social and educational contexts. Comparing Russia and Turkey makes it possible to identify both culturally specific and culturally universal patterns in the relationships between parental digital mediation and children’s digital play addiction tendencies.

The present study aimed to address two research questions: (1) What are the similarities and differences in the tendency toward digital play addiction among 5- to 6-year-old children and parental digital play mediation between Russia and Turkey? (2) How are parental digital mediation strategies related to children’s tendency toward digital play addiction in Russia and Turkey?

The study involved parents of 5- to 6-year-old children from Russia and Turkey. All children about whom parents reported were playing digital games. This age range was selected because, on the one hand, children at this age already play digital games, and on the other hand, they do not yet have their own mobile phones or tablets. Consequently, their digital activity is most often possible only with the participation of parents or other close adults (Teixeira et al., 2026). Thus, this age represents the most sensitive period for identifying the relationship between parental digital mediation and children’s tendency toward digital play addiction.

## Methods

### Participants

Through purposeful sampling method 452 parents of children 4–6 years old were selected for this study 226 from Russia and 226 from TurkeyTurkey. In both Russia and Turkey, 88% of the respondents were mothers (96% mothers in Russia and 80% in Turkey). Parents’ ages ranged from 25 to 47 years (*M* = 35.4, *SD* = 5.66). Regarding parental education level, in Russia 77% had college education or above, whereas in Turkey 46.5% had college education or above. The target children (about whom parents reported) were 50% male and 50% female in each country, with ages ranging from 5.0 to 6.9 years (M = 5.5, SD = 0.74).

### Procedure

Data collection was carried out by distributing a link to an online form directly to parents of 5- to 6-year-old children. Convenient preschools or kindergartens, mostly campus based programs in each country, were contacted by email to share the online forms. The study was anonymous. Parents were asked to respond specifically about their 5- to 6-year-old child (if they had more than one), and to indicate their own age, gender, education level, the child’s gender, and whether the child plays digital games. Subsequently, two questionnaires were provided for completion. Following preliminary data processing, the responses of 94 parents were excluded from the study because their children did not play digital games and/or did not meet the age criteria (younger than 5 or older than 6 years). The final dataset comprised responses from 452 parents.

This study was conducted in accordance with the ethical standards of the Declaration of Helsinki. This research was also approved by the Ethics Committee for Scientific Research of the Federal Scientific Center of Psychological and Multidisciplinary Research (approval No. 4, dated December 19, 2025).

### Measures

#### Digital play addiction tendency scale (DPAT)

The DPAT (Budak and Işıkoğlu, 2022) is a 20-item questionnaire rated on a 5-point Likert-type scale (1 = Never to 5 = Always). The original version comprises four subscales – Dissociation from Life, Conflict, Constant Play, and Reflection on Life – which are posited to measure distinct facets of children’s tendency toward digital play addiction. Total scores range from 20 to 100, with higher scores indicating stronger digital play addiction tendencies, classified from minimal to very high across established cut-off ranges.

#### Digital Play parental mediation scale (DPPM)

The DPPM (Budak and Işıkoğlu, 2022) is a 21-item questionnaire using the same 5-point response format. Its original structure includes four subscales: Active, Encouraging, Permissive, and Technical Parental Mediation, designed to assess the frequency of different parental mediation strategies.

Both scale items were translated into Russian by researchers. In order to evaluate language and cultural validity, the questionnaire was retranslated back to English using two bilingual people. The results verified the comparability of the items’ meanings across the languages (Douglas and Craig 2007).

### Data analysis

Before conducting cross-cultural comparisons, we tested the measurement invariance of both scales (DPAT and DPPM) across the Turkish and Russian samples using multi-group confirmatory factor analysis (MG-CFA) with the DWLS estimator in Jamovi 2.7.30. Because the items were measured on a five-point Likert scale and treated as ordinal indicators, threshold invariance was tested instead of intercept invariance. For the Digital Play Addiction Tendency Scale (DPAT), a four-factor structure (Dissociation from Life, Conflict, Constant Play, Reflection on Life) was specified. First, configural invariance was evaluated to determine whether the same four-factor structure was applicable across groups. The configural model demonstrated good fit to the data, χ^2^(320) = 610, CFI = 0.991, TLI = 0.990, RMSEA = 0.053, and SRMR = 0.069, indicating that the hypothesized factor structure was comparable across Turkish and Russian samples. Next, metric invariance was tested by constraining factor loadings to equality across groups. The metric model also demonstrated acceptable fit, χ^2^(335) = 663, CFI = 0.985, TLI = 0.984, RMSEA = 0.067, and SRMR = 0.078. Compared with the configural model, changes in fit indices were within recommended thresholds (ΔCFI = 0.006, ΔRMSEA = 0.014), supporting metric invariance. Finally, scalar (threshold) invariance was examined by additionally constraining item thresholds across groups. The scalar model showed good fit, χ^2^(358) = 698, CFI = 0.984, TLI = 0.985, RMSEA = 0.065, and SRMR = 0.071. Changes in fit indices relative to the metric model were negligible (ΔCFI = 0.001, ΔRMSEA = 0.002), indicating support for scalar invariance (See Table 1). Overall, the findings supported configural, metric, and scalar invariance across Turkish and Russian samples. Therefore, latent mean comparisons between the two countries were considered meaningful and statistically appropriate.

**Table 1:** Measurement invariance of the DPAT across Russian and Turkish samples.

Model	χ^2^	df	CFI	TLI	RMSEA [90% CI]	SRMR	ΔCFI	ΔRMSEA
1. Configural	610	320	0.991	0.990	0.053 [0.047, 0.060]	0.069	–	–
2. Metric	663	335	0.985	0.984	0.067 [0.060, 0.074]	0.078	0.006	0.014
3. Scalar (Threshold)	698	358	0.984	0.985	0.065 [0.058, 0.072]	0.071	0.001	0.002

ΔCFI and ΔRMSEA were calculated relative to the preceding model. Following Chen (2007), measurement invariance was supported when ΔCFI ≤ 0.010 and ΔRMSEA ≤ 0.015. For ordinal indicators, threshold invariance was evaluated instead of intercept invariance.

Again, measurement invariance of the DPPM was examined across Turkish and Russian children using multigroup confirmatory factor analysis with the diagonally weighted least squares (DWLS) estimator. Given the ordinal nature of the items, threshold invariance was assessed rather than intercept invariance. The configural model demonstrated acceptable fit to the data, χ^2^(344) = 670.48, CFI = 0.981, TLI = 0.979, RMSEA = 0.060, and SRMR = 0.085, indicating that the hypothesized factor structure was comparable across Turkish and Russian samples (See Table 2).

**Table 2:** Measurement invariance of the DPPM across Russian and Turkish samples.

Model	χ^2^	df	CFI	TLI	RMSEA [90% CI]	SRMR	ΔCFI	ΔRMSEA
1. Configural	670	344	0.981	0.979	0.060 [0.053, 0.067]	0.085	—	—
2. Metric	778	383	0.973	0.970	0.069 [0.062, 0.076]	0.093	0.008	0.009
3. Scalar (Threshold)	1,126	438	0.953	0.954	0.086 [0.080, 0.093]	0.090	0.020	0.017
4. Partial scalar (18, 19, 17, 12, 10)	933	433	0.963	0.963	0.074 [0.067, 0.081]	0.091	0.010	0.005

Measurement invariance was evaluated using changes in approximate fit indices. Following Chen (2007), invariance was considered supported when ΔCFI ≤ 0.010 and ΔRMSEA ≤ 0.015.

Next, metric invariance was tested by constraining factor loadings to equality across groups. The metric model also demonstrated acceptable fit, χ^2^(383) = 778.00, CFI = 0.973, TLI = 0.970, RMSEA = 0.069, and SRMR = 0.093. Changes in fit indices relative to the configural model were within recommended thresholds (ΔCFI = 0.008, ΔRMSEA = 0.009), supporting metric invariance across groups. These findings indicate that the relationships between the latent constructs and their indicators were equivalent across Turkish and Russian participants.

Subsequently, scalar (threshold) invariance was evaluated by constraining item thresholds across groups. The scalar model yielded χ^2^(438) = 1126.00, CFI = 0.953, TLI = 0.954, RMSEA = 0.086, and SRMR = 0.090. Relative to the metric model, the changes in fit indices exceeded recommended cut-off values (ΔCFI = 0.020, ΔRMSEA = 0.017), indicating that full scalar invariance was not supported.

To address this issue and justify subsequent latent mean comparisons, a partial scalar invariance model (Model 4) was estimated. Based on the modification indices and constraint score tests, the intercept constraints for five items that exhibited the highest cultural variance (DPPM10, DPPM12, DPPM17, DPPM18, and DPPM19) were systematically relaxed. The resulting partial scalar model achieved an acceptable and improved fit (χ^2^ = 933, df = 433, CFI = 0.963, TLI = 0.963, RMSEA = 0.074, SRMR = 0.091). Crucially, when compared against the metric model, the changes in approximate fit indices successfully met the rigorous cut-off criteria (ΔCFI = 0.010, ΔRMSEA = 0.005). Consequently, partial scalar invariance was supported, providing a solid methodological justification for conducting meaningful cross-cultural comparisons of latent means between the Russian and Turkish samples.

To address the second research question, bivariate associations between the DPAT and DPPM scales were assessed using Pearson’s correlation coefficient. Subsequently, separate linear regression models were built for each country, in which the DPAT scale scores served as the outcome variable and the four DPPM strategy scores served as predictors. Observed scores rather than latent multi-group structural equation modeling were utilized because full metric invariance was not established for the DPPM scale, making the structural comparison of latent paths across countries methodologically invalid.

To verify that the study was adequately powered to detect meaningful effects, an *a priori* power analysis was performed using G*Power (Version 3.1; Faul et al., 2009). For the linear regression models (fixed model, testing *R*^2^ deviation from zero), the parameters were set as follows: *f^2^* = 0.06 (between small and medium), *α* = 0.05, power = 0.80, with four predictors included. This analysis showed that a minimum of *N* = 204 participants was needed for each regression model. Consequently, the obtained sample sizes (*N* = 226 per country) can be considered sufficient detect the hypothesized effects without inflation of Type II error rates.

## Results

### Cross cultural comparisons of DPAT and DPPM in Russia and Turkey

Following the establishment of partial scalar invariance, latent mean comparisons were conducted across the Russian and Turkish samples to examine cross-cultural differences in children’s digital play addiction tendencies. The Russian sample was designated as the reference group (Group 1), with its latent means constrained to zero for all factors. The latent means of the Turkish sample (Group 2) were freely estimated and compared against the reference group.

As presented in Table 3, the latent mean comparison revealed statistically significant differences between the two countries across all subscales. Specifically, the Turkish sample exhibited significantly higher latent means compared to the Russian sample in all dimensions: Dissociation from Life (*γ* = 0.587, z = 6.295, *p* < 0.001), Conflict (γ = 0.633, *z* = 6.329, *p* < 0.001), Constant Play (γ = 0.386, *z* = 3.829, *p* < 0.001), and Reflection on Life (γ = 0.685, *z* = 5.437, *p* < 0.001). These findings indicate that the Turkish participants demonstrated a systematically higher tendency toward digital play addiction across all specified psychological domains than their Russian counterparts.

**Table 3:** Comparison of DPAT and DPPM scores in children from Russia and Turkey.

Factor	Country	Latent mean estimate	*z*-value	*p*	Effect size (*d*)
DPAT-dissociation from life	Russia (Ref)	0.000	–	–	
	Turkey	0.587	6.295	<0.001	0.59
DPAT-conflict	Russia (Ref)	0.000	–	–	
	Turkey	0.633	6.329	<0.001	0.63
DPAT-constant play	Russia (Ref)	0.000	–	–	
	Turkey	0.386	3.829	<0.001	0.39
DPAT reflection on life	Russia (Ref)	0.000	–	–	
	Turkey	0.685	5.437	<0.001	0.69
DPPM – active	Russia (Ref)	0.000	–	–	–
	Turkey	−0.062	−0.628	0.530	−0.06
DPPM – encouraging	Russia (Ref)	0.000	–	–	–
	Turkey	−0.258	−2.004	0.045	−0.26
DPPM- permissive	Russia (Ref)	0.000	–	–	–
	Turkey	−0.131	−1.012	0.312	−0.13
DPPM-technical	Russia (Ref)	0.000	–	–	–
	Turkey	−0.197	−1.704	0.088	−0.20

Ref, Reference group. Latent means for the reference group (Russia) were constrained to zero. Positive estimates indicate higher latent mean scores among Turkish children relative to Russian children. Effect sizes (d) are reported using standardized latent mean differences and may be interpreted according to Cohen’s (1988) guidelines: 0.20 = small, 0.50 = medium, and 0.80 = large effect.

As presented in Table 3, the latent mean comparison for DPPM dimensions revealed partially significant differences between the two countries. The Turkish sample exhibited significantly lower latent mean only in the Encouraging dimension compared to the Russian sample (γ = −0.258, *z* = −2.004, *p* = 0.045, *d* = −0.26). No statistically significant differences were observed in the Active dimension (γ = −0.062, *z* = −0.628, *p* = 0.530, *d* = −0.06), Permissive dimension (γ = −0.131, *z* = −1.012, *p* = 0.312, *d* = −0.13), or Technical dimension (γ = −0.197, *z* = −1.704, *p* = 0.088, *d* = −0.20), although the latter approached marginal significance.

### Relationships between children’s digital play addiction tendency and digital parental mediation in Russia and Turkey

Associations between children’s digital play addiction tendency scores and digital parental mediation were examined separately for Russia and Turkey (Tables 4, 5). In both Russia and Turkey, Encouraging Parental Mediation and Permissive Parental Mediation were positively associated with all aspects of children’s digital play addiction tendency. Active Parental Mediation in Turkey was negatively associated with all aspects of children’s digital play addiction tendency except Reflection on Life, whereas in Russia it was negatively associated with all aspects except Dissociation from Life and Reflection on Life. Technical Parental Mediation in Turkey was negatively associated with Constant Play, whereas in Russia it was negatively associated with Dissociation from Life and Conflict.

**Table 4:** Correlation between the scales of the DPAT and DPPM questionnaires in Russia.

Scale	Dissociation from life	Conflict	Constant play	Reflection on life	Total score
Active parental mediation	−0.088	−0.175*	−0.185**	−0.006	−0.165*
Encouraging parental mediation	0.233**	0.318***	0.502***	0.260***	0.411***
Permissive parental mediation	0.233**	0.264***	0.450***	0.297***	0.382***
Technical parental mediation	−0.177*	−0.188*	−0.133	−0.077	−0.175*

*p < 0.05; **p < 0.01; ***p < 0.001.

**Table 5:** Correlation between the scales of the DPAT and DPPM questionnaires in Turkey.

Scale	Dissociation from life	Conflict	Constant play	Reflection on life	Total score
Active parental mediation	−0.200**	−0.190**	−0.356***	−0.084	−0.275***
Encouraging parental mediation	0.383***	0.449***	0.545***	0.293***	0.538***
Permissive parental mediation	0.233**	0.214**	0.384***	0.215**	0.327***
Technical parental mediation	−0.093	−0.060	−0.184**	−0.108	−0.138*

*p < 0.05; **p < 0.01; ***p < 0.001.

The differing correlational structure between the DPAT and DPPM prompted us to build regression models in which DPAT subscale scores served as dependent variables, and DPPM subscale scores served as candidate predictors, separately for Russia and Turkey. As a result, eight models were constructed (Tables 6, 7). Applying the Bonferroni correction, only results with *p* < 0.006 were considered significant.

**Table 6:** Regression models for different aspects of children’s play addiction to gadgets in Russia.

Predictor	Outcome	B	SE	*β*	*t*	*p*	95% confidence interval	
							Lower	Upper
(Intercept)	Dissociation from life *F*(4, 221) = 5.037, *p* < 0.001	7.808	1.717		4.546	<0.001	4.422	11.195
APM		−0.207	0.373	−0.041	−0.553	0.581	−0.942	0.529
EPM		1.101	0.648	0.139	1.700	0.091	−0.176	2.379
PPM		0.817	0.442	0.152	1.849	0.066	−0.054	1.689
TPM		−0.309	0.185	−0.124	−1.675	0.095	−0.673	0.055
(Intercept)	Conflict *F*(4, 221) = 9.004, *p* < 0.001	9.520	1.763		5.399	<0.001	6.043	12.997
APM		−0.783	0.383	−0.148	−2.044	0.042	−1.539	−0.028
EPM		1.856	0.665	0.220	2.790	0.006	0.545	3.168
PPM		0.856	0.454	0.150	1.886	0.061	−0.039	1.750
TPM		−0.231	0.189	−0.087	−1.221	0.224	−0.605	0.142
(Intercept)	Constant play *F*(4, 221) = 25.418, *p* < 0.001	5.597	1.349		4.149	<0.001	2.937	8.257
APM		−0.895	0.293	−0.195	−3.052	0.003	−1.473	−0.317
EPM		2.419	0.509	0.332	4.752	<0.001	1.415	3.423
PPM		1.392	0.347	0.282	4.009	<0.001	0.707	2.076
TPM		−0.043	0.145	−0.019	−0.296	0.767	−0.329	0.243
(Intercept)	Reflection on life *F*(4, 221) = 5.897, *p* < 0.001 *R*^2^ = 0.105	3.537	0.950		3.725	<0.001	1.665	5.410
APM		0.023	0.206	0.008	0.114	0.910	−0.383	0.430
EPM		0.603	0.358	0.136	1.682	0.094	−0.104	1.309
PPM		0.652	0.244	0.217	2.667	0.004	0.170	1.134
TPM		−0.083	0.102	−0.060	−0.818	0.414	−0.285	0.118

APM, active parental mediation; EPPM, encouraging parental mediation; PPM, permissive parental mediation; TPM, technical parental mediation.

**Table 7:** Regression models for different aspects of children’s addiction to gadgets in Turkey.

Predictor	Outcome	B	SE	*β*	*t*	*p*	95% confidence interval	
							Lower	Upper
(Intercept)	Dissociation from life *F*(4, 221) = 9.684, *p* < 0.001 *R*^2^ = 0.149	7.804	3.162		2.468	0.014	1.571	14.036
APM		−0.130	0.564	−0.018	−0.231	0.818	−1.242	0.982
EPM		2.631	0.569	0.356	4.623	<0.001	1.509	3.752
PPM		0.365	0.542	0.049	0.673	0.502	−0.703	1.432
TPM		0.097	0.360	0.019	0.270	0.787	−0.612	0.806
(Intercept)	Conflict *F*(4, 221) = 14.239, *p* < 0.001 *R*^2^ = 0.205	4.599	2.202		2.089	0.038	0.260	8.938
APM		0.088	0.393	0.017	0.223	0.824	−0.686	0.862
EPM		2.481	0.396	0.466	6.263	<0.001	1.700	3.262
PPM		0.048	0.377	0.009	0.127	0.899	−0.695	0.791
TPM		0.185	0.250	0.051	0.739	0.461	−0.308	0.679
(Intercept)	Constant play *F*(4, 221) = 26.294, *p* < 0.001 *R*^2^ = 0.322	5.504	1.903		2.892	0.004	1.754	9.255
APM		−0.496	0.340	−0.102	−1.462	0.145	−1.166	0.173
EPM		2.142	0.342	0.429	6.255	<0.001	1.467	2.817
PPM		0.713	0.326	0.143	2.186	0.030	0.070	1.355
TPM		0.045	0.216	0.013	0.206	0.837	−0.382	0.471
(Intercept)	Reflection on life *F*(4, 221) = 6.265, *p* < 0.001 *R*^2^ = 0.102	3.255	1.390		2.341	0.020	0.515	5.996
APM		0.370	0.248	0.119	1.492	0.137	−0.119	0.859
EPM		0.910	0.250	0.287	3.635	<0.001	0.416	1.403
PPM		0.314	0.238	0.099	1.320	0.188	−0.155	0.784
TPM		−0.132	0.158	−0.061	−0.833	0.406	−0.443	0.180

APM, active parental mediation; EPPM, encouraging parental mediation; PPM, permissive parental mediation; TPM, technical parental mediation.

For Russia, all regression models were statistically significant (all *p* < 0.001).

- *Dissociation from Life* (*R*^2^ = 0.091): No digital parental mediation strategy was a significant predictor of this aspect of digital play addiction.
- *Conflict* (*R*^2^ = 0.151): Encouraging Parental Mediation was the only significant predictor (*β* = 0.220, *p* = 0.006).
- *Constant Play* (*R*^2^ = 0.335): Both Encouraging Parental Mediation (*β* = 0.332, *p* < 0.001) and Permissive Parental Mediation (*β* = 0.282, *p* < 0.001) were significant positive predictors. Additionally, Active Parental Mediation was a negative predictor (*β* = −0.195, *p* = 0.003).
- *Reflection on Life* (*R*^2^ = 0.105): Permissive Parental Mediation was a significant positive predictor (*β* = 0.217, *p* = 0.004).
- For Turkey, all regression models were statistically significant (all *p* < 0.001).
- *Dissociation from Life* (*R*^2^ = 0.149): Encouraging Parental Mediation was the only significant predictor (*β* = 0.356, *p* < 0.006).
- *Conflict* (*R*^2^ = 0.205): Encouraging Parental Mediation was a significant positive predictor (*β* = 0.446, *p* < 0.001).
- *Constant Play* (*R*^2^ = 0.332): Encouraging Parental Mediation was a significant positive predictor (*β* = 0.429, *p* < 0.001).
- *Reflection on Life* (*R*^2^ = 0.102): Encouraging Parental Mediation was the only significant positive predictor (*β* = 0.287, *p* < 0.001).

For all significant regression models, *R*^2^ > 0.09, implying that *f*^2^ > 0.10 for these models. This exceeds the effect size assumed in the *a priori* power analysis (*f*^2^ = 0.06).

Across both countries, Encouraging Parental Mediation emerged as the most consistent predictor of children’s digital play addiction tendencies. In Turkey, it significantly predicted all four dimensions of digital play addiction, whereas in Russia it significantly predicted Conflict and Constant Play. These findings suggest that parental encouragement of digital play may represent a cross-culturally robust risk factor associated with elevated levels of children’s digital play addiction tendencies.

## Discussion

This study explores children’s digital play addiction tendencies and parental mediation strategies across two distinct national contexts. Although the research is situated within two specific nations, digital play is a global phenomenon; consequently, these findings offer valuable insights into addiction tendencies and parental mediation across diverse cultural landscapes. In light of the empirical findings and existing literature, it can be concluded that digital play addiction is an increasingly critical issue, and that parental mediation plays an essential role in influencing these tendencies in young children.

Comparative analysis reveals that Turkish children exhibited significantly higher digital addiction tendencies across all subscales than their Russian peers. The observed differences ranged from small-to-moderate to moderate effect sizes, indicating that the country differences were not only statistically significant but also practically meaningful. Specifically, preschool enrollment in Turkey is relatively high, reaching 82% among five-year-olds; however, these programs are typically structured as half-day sessions (Millî Eğitim Bakanlığı (MEB), 2025). Consequently, these children possess a substantial amount of unstructured time at home, which is frequently occupied by digital device usage and gaming (Kahriman and Borg, 2024). In contrast, approximately 93% of Russian children attend full-day kindergarten on weekdays (typically from 8:00–9:00 a.m. to 5:00–7:00 p.m.), providing a highly structured environment where interaction with digital devices is strictly minimized. Despite the greater expression of almost all aspects of digital play addiction among Turkish children, differences in the frequency of conflicts arising from depriving the child of the opportunity to play digital games (digital tantrums) are questionable. This suggests that even the shorter amount of time spent playing digital games (among Russian children) is sufficiently attractive that children find it very difficult to disengage from this activity calmly (Soldatova et al., 2024). It is also conceivable that the observed differences are partly driven by the fact that digital technologies are more widely utilized in preschool educational settings in Turkey than in Russia (Kahriman and Borg, 2024; Kholodova et al., 2025).

Comparing parental mediation strategies of parents from both countries indicated that Turkish parents showed significantly lower latent means on the Encouraging subscale compared to their Russian counterparts (*γ* = −0.258, *p* = 0.045). No significant differences were found for the Active, Permissive, or Technical subscales of DPPM. This subtle difference in explicit encouragement presents an intriguing cultural nuance. First, the Encouraging dimension may capture distinct normative expectations and social desirability biases regarding the active promotion of digital engagement in early childhood. Russian preschoolers may navigate a social ecology where digital exploration is more explicitly scaffolded and normalized by the immediate environment. Conversely, while Turkish children demonstrate elevated digital play addiction tendencies, their parents may extend less explicit encouragement. This pattern likely underscores a cultural divergence in the socialization of technology: Turkish parents might perceive digital play through a more restrictive or problematic lens despite their children’s high engagement, whereas Russian parents may normalize or actively integrate it into daily routines. In such contexts, digital devices frequently function as contemporary “digital pacifiers” to manage children’s behavior during demanding situational transitions, such as travel or mealtimes (Brauchli et al., 2024b).

Beyond these differences in latent scores, we should also consider the broader similarities in how parents from both cultures approach digital mediation (Savchenko et al., 2025). Interestingly, in both Russian and Turkish families, Active and Technical mediation emerged as the most commonly used strategies. These are generally seen as positive, proactive approaches to guiding children’s digital experiences (Işıkoğlu et al., 2025). What stands out here is that parents from two quite different cultural backgrounds ended up with remarkably similar preferences. They both seem to rely on the same types of strategies when it comes to managing their preschoolers’ screen time. This is worth paying attention to because, despite ongoing concerns from developmental psychologists about how much parents really understand digital technologies (Klopotova et al., 2022), this study tells a slightly different story. Parents seem to have an intuitive grasp of how to guide their young children’s digital experiences in a way that supports healthy development.

However, given the cross-sectional nature of this study, the causal direction of the association between parental mediation and children’s digital play behavior remains ambiguous. It is plausible that child-driven effects exist, such that children’s play behavior elicits certain parental responses. Nonetheless, we contend that at the initial stages of digital engagement — specifically among children aged 5 to 6 years — parental mediation strategies are more likely to precede and shape children’s digital behavior, rather than being predominantly reactive to it (Çalhan and Göksu, 2024; Soldatova and Teslavskaya, 2019).

Regarding the prediction of children’s digital play addiction tendencies through parental digital mediation strategies in Russia and Turkey, the results indicated that all examined dimensions of digital play addiction are largely associated with how frequently parents utilize encouraging parental mediation. These findings align closely with a similar study conducted in Portugal (Teixeira et al., 2026), which likewise identified encouraging parental mediation as a notable risk factor for the development of digital play addiction tendencies in young children. Although separate regression analyses suggested somewhat different patterns across countries, the present study did not statistically compare regression coefficients between groups. Therefore, these differences should be interpreted cautiously.

For Russia, the results are less homogeneous. Dissociation from Life was not associated (at a level greater than 0.20 in absolute value) with any digital parental mediation strategy. It is therefore unsurprising that no strategy was a predictor of this aspect of addiction tendency. We attribute this to the generally lower expression of this aspect among Russian children compared to Turkish children, which in turn may be explained by Russian parents’ greater emphasis on controlling the routine aspects of the child’s life (Soldatova and Teslavskaya, 2019). The expression of Conflict regarding the cessation of gameplay was found to be partially determined by the frequency of Encouraging Parental Mediation, which coincides with the results obtained for Turkey and underscores the culturally universal role of using this strategy in pronounced conflicts with children regarding digital devices. Constant Play among Russian children was positively related to the frequency of Encouraging and Permissive Parental Mediation and negatively linked to the frequency of Active Parental Mediation. This result is particularly interesting because, for this aspect of children’s tendency toward digital play addiction, a protective role of Active Parental Mediation was identified, confirming the importance of discussing children’s digital play activity with them to minimize risks (Lwin et al., 2008). As for the expression of Reflection on Life among 5- to 6-year-old children in Russia, the frequency with which parents resort to Permissive Mediation turned out to be a predictor. This result appears logical, as Permissive Mediation implies that the child chooses content independently, and these preferences may carry over into non-digital play and other areas of the child’s life.

Taken together, the findings highlight that not all parental mediation strategies operate in the same manner. Whereas encouraging and permissive forms of mediation were generally associated with higher levels of digital play addiction tendencies, active mediation showed evidence of a potentially protective role. These findings emphasize the importance of considering both the quantity and quality of parental involvement in children’s digital activities.

### Limitations of the study

Several limitations should be considered when interpreting the findings of this study. First, the study employed a cross-sectional design. Therefore, although parental mediation strategies were associated with children’s digital play addiction tendencies, causal inferences cannot be made. It remains unclear whether specific parental mediation strategies contribute to higher levels of digital play addiction tendencies or whether children’s existing digital play behaviors influence parental mediation practices. Longitudinal and experimental studies are needed to clarify the directionality of these relationships. Second, all data were collected through parent-report questionnaires. Consequently, the findings may be affected by social desirability bias, recall bias, and parents’ subjective perceptions of their children’s digital play behaviors. Future studies would benefit from incorporating multiple informants and objective indicators, such as direct observations or digital usage records. Third, several contextual factors that may influence both parental mediation practices and children’s digital play addiction tendencies were not included in the present study. Variables such as family income, parental education, parental digital literacy, household composition, children’s daily screen time, and preschool attendance patterns may contribute to the observed relationships and should be examined in future research (Denisenkova and Taruntaev, 2023; Nikolaeva et al., 2023; Kesicioğlu, 2026). Finally, participants were recruited through an online survey procedure, which may limit the representativeness of the sample. Families with greater access to digital technologies and higher levels of digital engagement may have been more likely to participate, potentially limiting the generalizability of the findings. Moreover, the inclusion of observational measures of children’s digital play behavior and child-directed interviews could have further enhanced the validity and depth of our conclusions.

## Conclusion

The present study contributes to the growing body of research on children’s digital play by examining digital play addiction tendencies and parental mediation practices across two distinct cultural contexts, Turkey and Russia. The findings revealed that Turkish children exhibited higher levels of digital play addiction tendencies than Russian children across all dimensions of the construct, suggesting that cultural and contextual factors may shape children’s engagement with digital games during early childhood. The study also demonstrated that parental mediation strategies are differentially associated with children’s digital play addiction tendencies. Across both countries, Encouraging Parental Mediation emerged as the most consistent correlate of higher levels of digital play addiction tendencies, whereas Active Parental Mediation showed evidence of a potentially protective role, particularly in relation to children’s tendency toward constant play. These findings indicate that parental involvement in children’s digital activities is not uniformly beneficial and that the specific type of mediation strategy employed may influence children’s digital experiences in different ways.

From a practical perspective, the results highlight the importance of supporting parents in developing effective mediation practices that promote healthy digital engagement while minimizing the risks associated with excessive digital play. Programs aimed at increasing parents’ awareness of the potential consequences of encouraging excessive digital game use may be particularly valuable during the preschool years. The findings contribute to the growing cross-cultural literature by demonstrating that parental mediation strategies may function differently depending on the type of mediation employed and the broader cultural context.

## Data Availability

The raw data supporting the conclusions of this article will be made available by the authors, without undue reservation.
